# A beneficial tumor microenvironment in oropharyngeal squamous cell carcinoma is characterized by a high T cell and low IL-17^+^ cell frequency

**DOI:** 10.1007/s00262-016-1805-x

**Published:** 2016-02-22

**Authors:** Simone Punt, Emilie A. C. Dronkers, Marij J. P. Welters, Renske Goedemans, Senada Koljenović, Elisabeth Bloemena, Peter J. F. Snijders, Arko Gorter, Sjoerd H. van der Burg, Robert J. Baatenburg de Jong, Ekaterina S. Jordanova

**Affiliations:** 1 Department of Pathology, Leiden University Medical Center (LUMC), P.O. Box 9600, 2300 RC Leiden, The Netherlands; 2 Department of Otorhinolaryngology and Head and Neck Surgery, Erasmus University Medical Center, Rotterdam, The Netherlands; 3 Department of Clinical Oncology, LUMC, Leiden, The Netherlands; 4 Department of Pathology, Erasmus University Medical Center, Rotterdam, The Netherlands; 5 Department of Pathology, VU Medical Center (VUMC), Amsterdam, The Netherlands; 6 Department of Oral and Maxillofacial Surgery/Oral Pathology, Academic Centre for Dentistry, Amsterdam, The Netherlands; 7 Center for Gynecological Oncology Amsterdam, VUMC, Amsterdam, The Netherlands

**Keywords:** Head and neck cancer, Tumor microenvironment, T cell, Th17 cell, IL-17, Treg

## Abstract

Patients with HPV-positive oropharyngeal squamous cell carcinomas (OPSCCs) have a better prognosis than patients with non-HPV-induced OPSCC. The role of the immune response in this phenomenon is yet unclear. We studied the number of T cells, regulatory T cells (Tregs), T helper 17 (Th17) cells and IL-17^+^ non-T cells (mainly granulocytes) in matched HPV-positive and HPV-negative OPSCC cases (*n* = 162). Furthermore, the production of IFN-γ and IL-17 by tumor-infiltrating T cells was analyzed. The number of tumor-infiltrating T cells and Tregs was higher in HPV-positive than HPV-negative OPSCC (*p* < 0.0001). In contrast, HPV-negative OPSCC contained significantly higher numbers of IL-17^+^ non-T cells (*p* < 0.0001). Although a high number of intra-tumoral T cells showed a trend toward improved survival of all OPSCC patients, their prognostic effect in patients with a low number of intra-tumoral IL-17^+^ non-T cells was significant with regard to disease-specific (*p* = 0.033) and disease-free survival (*p* = 0.012). This suggests that a high frequency of IL-17^+^ non-T cells was related to a poor immune response, which was further supported by the observation that a high number of T cells was correlated with improved disease-free survival in the HPV-positive OPSCC (*p* = 0.008). In addition, we detected a minor Th17 cell population. However, T cells obtained from HPV-positive OPSCC produced significantly more IL-17 than those from HPV-negative tumors (*p* = 0.006). The improved prognosis of HPV-positive OPSCC is thus correlated with higher numbers of tumor-infiltrating T cells, more active Th17 cells and lower numbers of IL-17^+^ non-T cells.

**Electronic supplementary material:**

The online version of this article (doi:10.1007/s00262-016-1805-x) contains supplementary material, which is available to authorized users.

## Introduction

Oropharyngeal squamous cell carcinoma (OPSCC) can be divided into subtypes with different etiologies, one subtype due to alcohol and tobacco use and another due to persistent infection with high-risk human papillomavirus (HPV) [1, 2]. The incidence of OPSCC and the prevalence of HPV-associated tumors are increasing in Europe and the USA [3–7]. The reported proportion of HPV-positive OPSCC ranges from 20 to 90 %. This high variation between studies may be related to the time period in which HPV prevalence was investigated as well as to the lack of a standardized HPV detection assay [8]. Remarkably, patients with HPV-positive OPSCC have a significantly better prognosis than patients with non-HPV-induced tumors [8–11]. However, heavy smoking habits seem to undo the beneficial effect of HPV positivity on survival [12].

A different type of cancer, arising in the cervix uteri, is practically always initiated by a persistent HPV infection [13]. From studies on cervical HPV infections, HPV is known to be cleared in over 90 % of cases [14]. In case of cervical cancer development, the tumor cells are thought to manipulate the immune response such that it facilitates tumor growth [15]. In addition, the immune response present in the tumor microenvironment is critical for clinical outcome. As OPSCC can be divided in virally induced and non-virally induced subtypes, this tumor type provides a means to study the relationship between the immune response and clinical outcome as a function of viral etiology. A high frequency of intratumoral CD8^+^ cytotoxic T cells has been found to be correlated with improved survival in OPSCC [16]. However, data are still limited for other T cell subsets in OPSCC, including regulatory T cells (Tregs).

The role of Tregs in cancer in general seems to be context and tumor type dependent [17], with correlations reported between a high Treg frequency and poor prognosis [18–20] but also improved prognosis [21–24]. The role of T helper 17 (Th17) cells and other IL-17 expressing cells is unclear, with contradictory functions attributed to this cell type in cancer [25]. We have recently shown that Th17 cells tend to be correlated with improved survival, while total IL-17, predominantly expressed by granulocytes, correlated with poor survival in cancer patients [26, 27].

The aim of this study was to elucidate the role of the immune response in virally induced versus non-virally induced OPSCC. We determined the distribution of intra-epithelial and stromal T cells, Tregs, Th17 and IL-17^+^ non-T cells with regard to HPV status in a large series of OPSCC cases and analyzed the correlations with patient survival. The IL-17^+^ non-T cells were included in these analyses, because we previously found that on average only 6 % of the tumor-infiltrating IL-17^+^ cells in head and neck cancer were Th17 cells, while 45 % were granulocytes [26]. In addition, the production of IFN-γ and IL-17 by tumor-infiltrating lymphocytes upon mitogenic stimulation was compared in HPV-positive and HPV-negative tumors. Because of the differences in survival based on HPV status, the differences in immune response between OPSCC groups may indicate markers of a beneficial immune response. We hypothesized that HPV-positive tumors are characterized by a different quantity and composition of immune cell infiltrates. We expect that total T cells and Th17 cells are correlated with improved clinical outcome, while Tregs and IL-17^+^ non-Th17 cells are correlated with poor outcome for patients.

## Materials and methods

### Patient material

For this study, we searched the hospital-based cancer registry Oncology Documentation (ONCDOC) of the LUMC for all primary oropharyngeal tumors, diagnosed between 1970 and 2011. Trained data managers scored all patient, treatment and follow-up data. These data were retrieved from the patients’ medical record and hospital-based data system. ONCDOC also performs an independent and active follow-up. Formaldehyde fixed, paraffin-embedded (FFPE) pretreatment tumor samples from 341 patients were obtained from the archives of the Pathology Department of the LUMC. A dedicated pathologist (Senada Koljenović) analyzed the tumor samples for the presence of malignant cells. Thirty tumor samples (8.8 %) were excluded for further analysis due to the absence of malignant cells. All included patients were treated following standard guidelines that applied in the year of diagnosis. Patient and tumor characteristics are listed in Table 1. The median follow-up time of the 162 patients selected for final analysis was 37 months. Patient samples were handled according to the medical ethical guidelines described in the Code of Conduct for Proper Secondary Use of Human Tissue of the Dutch Federation of Biomedical Scientific Societies (www.federa.org).

**Table 1:** Patient and tumor characteristics

Clinicopathological parameter	Category	HPV-negative tumors (%) (*n* = 99)	HPV-positive tumors (%) (*n* = 63)
Age	Median (years)	60	57
	Range (years)	41–86	43–90
Sex	Female	32 (32)	25 (40)
	Male	67 (68)	38 (60)
Tumor location	Base of tongue	23 (23)	16 (25)
	Tonsil	25 (25)	31 (49)
	Tonsillar fossa	26 (26)	9 (14)
	Oropharyngeal wall	13 (13)	4 (6)
	Soft palate	8 (8)	1 (2)
	Vallecula	1 (1)	2 (3)
	Uvula	3 (3)	0 (0)
Tumor morphology	Squamous cell (unspecified)	35 (35)	32 (51)
	Keratinizing squamous cell	45 (45)	14 (22)
	Non-keratinizing squamous large cell	16 (16)	16 (25)
	Papillary squamous cell	2 (2)	1 (2)
	Squamous spindle cell	1 (1)	0 (0)
TNM stage^1^	T1	16 (16)	19 (30)
	T2	33 (33)	27 (43)
	T3	26 (26)	13 (21)
	T4	24 (24)	4 (6)
	N0	38 (38)	9 (14)
	N1	22 (22)	13 (21)
	N2	33 (33)	38 (60)
	N3	6 (6)	3 (5)
	M0	97 (98)	62 (98)
	M1	2 (2)	1 (2)
Local recurrence	No	83 (84)	55 (87)
	Yes	16 (16)	8 (13)
Regional recurrence	No	82 (83)	57 (90)
	Yes	17 (17)	6 (10)
Distant metastasis	No	84 (85)	57 (90)
	Yes	15 (15)	6 (10)
Deceased	No	29 (29)	45 (71)
	Yes	70 (71)	18 (29)
Follow-up time	Median (months)	28	55
Prior tumor	No	93 (94)	55 (87)
	Yes	6 (6)	8 (13)
Smoking	Never	9 (9)	19 (30)
	Medium (1–24 PY)	8 (8)	11 (18)
	Heavy (>24 PY)	33 (33)	15 (24)
	Unknown	49 (50)	18 (28)

^1^Clinical TNM classification of the tumor size (*T*) and the involvement of regional lymph nodes (*N*) and distant metastases (*M*)

### p16 and HPV detection

FFPE tumor specimens from 311 patients were cut into 4-µm-thick sections, deparaffinized and stained for p16 (INK4A; Roche MTM Laboratories AG, Heidelberg, Germany) using a fully automated Ventana BenchMark ULTRA Stainer (Ventana, Tucson Arizona, USA) according to the manufacturers’ instructions. Binding of peroxidase-coupled antibodies was visualized using 3,3′-diamino-benzidine-tetrahydrochloride (DAB). Slides were counterstained with hematoxylin. P16 immunostained samples were scored independently by two dedicated pathologists (Senada Koljenović, Elisabeth Bloemena). The tumor samples were scored as ‘p16 positive’ when >70 % of the tumor cells showed both nuclear and cytoplasmic staining.

High-risk HPV DNA detection was performed on the p16-positive cases. DNA was extracted from all p16 positive cases using an automated silica-based extraction system, and PCR was performed using the HPV-Risk assay (Self-Screen BV, Amsterdam, the Netherlands) [28]. The HPV-Risk assay is a novel real-time PCR assay targeting the E7 region of 15 high-risk HPV types (i.e., HPV 16, 18, 31, 33, 35, 39, 45, 51, 52, 56, 58, 59, 66, 67 and 68) and provides additional genotype information for HPV 16 and HPV 18. The HPV-Risk assay is clinically validated and meets the cross-sectional clinical and reproducibility criteria of the international guidelines for HPV test requirements.

### Matching

All p16 positive cases (*n* = 94) were included in this study as well as *n* = 94 p16 negative cases that were matched for tumor T stage, N stage, location, patient gender and decennium of diagnosis. Hence, a subset of *n* = 188 out of *n* = 311 patients was selected for the present study.

### Immunofluorescent stainings

Part of the selected *n* = 188 tumor samples could not be analyzed due to insufficient tumor material to obtain at least one microscopic image (*n* = 26). Triple immunofluorescent staining for CD3 (ab828, Abcam, Cambride, UK), FoxP3 (ab20034, Abcam) and IL-17 (AF-317-NA, R&D Systems, Abingdon, UK) was performed on 162 tumor samples as described before [29]. These comprised 86 p16 positive and 76 p16 negative tumors. Images were obtained using an LSM700 confocal laser scanning microscope containing an LCI Plan-Neofluar 25×/0.8 Imm Korr DIC M27 objective (Zeiss, Göttingen, Germany). One to four random images sampled a total vital tumor (epithelium + stroma) area of up to 1.0 mm^2^. Total tumor epithelium and stroma surface area and double or triple positivity of cells were determined in each image using LSM Image Browser (version 4.2.0.121, Zeiss). Single-, double- and triple-positive cells were scored separately in the tumor epithelial and stromal areas using ImageJ version 1.47 (http://rsb.info.nih.gov/ij). Cells within blood vessels and largely autofluorescent areas were not scored.

### TIL isolation and cytokine analysis

Fresh OPSCC tissue was cut into small pieces of ~1 mm^3^ and cultured in IMDM (Lonza), supplemented with 10 % human AB serum (Life Technology) and two–three times a week 1000 IU/mL IL-2 (Novartis Aldesleukin). TIL were cultured for two–four weeks to obtain sufficient cells for testing their response to PHA stimulation (in triplicate wells). Unstimulated T cells were used as a negative control. Supernatant (50 µl/well) was harvested after 4 days of stimulation and used for cytokine analysis. The production of IFN-γ (Sanquin) and IL-17A (eBiosciences) was analyzed according to the manufacturers’ ELISA kit guidelines.

### Statistical analysis

Statistical analyses were performed using SPSS version 20.0 (IBM Corp., Armonk, USA) and R version 3.1.1. (packages: foreign, mice, rms, survival). Differences in the numbers of positive cells between patient groups were tested using the Wilcoxon Mann–Whitney tests. Correlations (*r*) between cell frequencies were tested using the Spearman’s rank correlation rho test. For each disease-free (time from diagnosis until local or distant recurrence or death to disease) and disease-specific (time from diagnosis until death to disease) survival, Kaplan–Meier curve generation and log rank analysis, the cell numbers were divided into four equal quartiles and the lowest quartile (low frequency) was compared with the other quartiles (high frequency). For comparisons based on a ratio or other combination of cell frequencies, patients were divided into a high and low group based on the median. Missing values for the variable ‘smoking status’ were handled by performing multiple imputation using the package ‘mice’ in *R*. All variables included in Table 1 were used for imputation. *N* = 5 imputations were performed, and the pooled imputed data were used in multivariate analyses. Multivariate analysis was performed using Cox proportional hazard regression analysis. All tests were two-sided, and *p* values below 0.05 were considered statistically significant.

## Results

### HPV analysis

Of the initial 311 tumor samples that were evaluated for HPV status, 94 (30 %) were scored ‘p16 positive.’ The inter-observer variability between the scoring of all tumor samples by two pathologists was 0.867 (kappa statistic, *p* < 0.001). Of the p16 positive cases, 70 (74.4 %) contained high-risk HPV DNA, of which 63 (90 %) were HPV 16 positive, and 7 (10 %) contained HPV 18 or other types of high-risk HPV. The variability between the p16 and PCR analyses was 0.774 (kappa statistic, *p* < 0.001). After matching all p16 positive cases (*n* = 94) for tumor T stage, N stage, location, patient gender and decennium of diagnosis with an equal amount of p16 negative cases, only 162 samples were suitable for further analysis by immunofluorescence. Of this subset of tumor samples, 86 were p16 positive and 76 were p16 negative. A proportion of 73.3 % (*n* = 63) of the p16 positive cases contained high-risk HPV DNA. Only these 63 cases were taken into account as HPV positive in further analysis. As a result, p16 positive but HPV DNA negative cases were considered HPV negative (*n* = 21). N stage, location of tumor, patient gender, patient age and level of comorbidity were not significantly different between the HPV-positive and HPV-negative cases. A higher T stage was observed in the HPV-negative patients, which might be due to the p16 positive HPV-negative patients to be considered HPV negative in the final analysis. However, adding T stage to the multivariate analyses did not influence the significance of the results.

### HPV-positive tumors are more heavily infiltrated by T cells and less by IL-17^+^ non-T cells

**Fig. 1:**
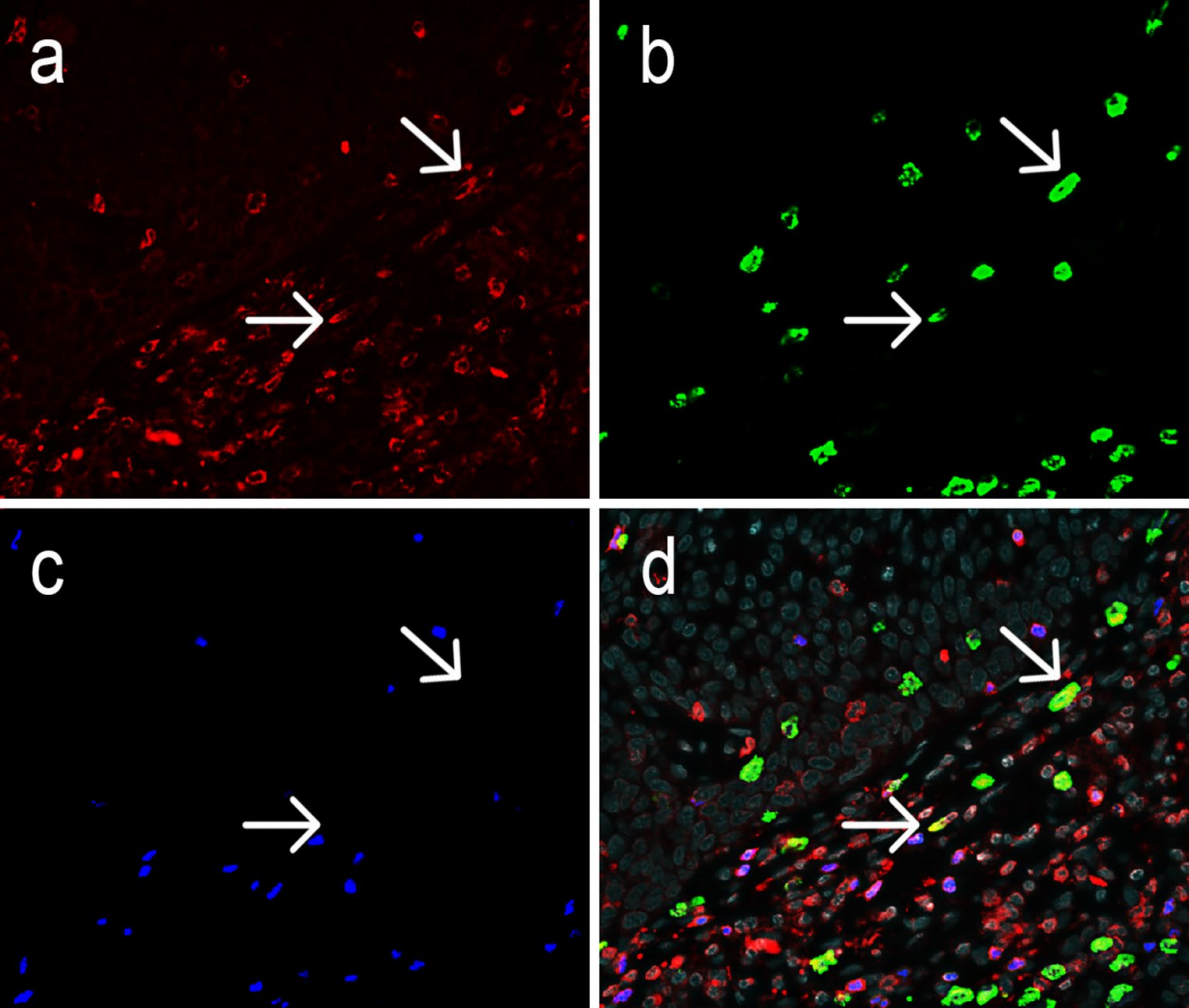
Representative image of an oropharyngeal cancer specimen stained by triple immunofluorescence for CD3 (**a**, *red*), IL-17 (**b**, *green*) and FoxP3 (**c**, *blue*), with the combined stainings together with DAPI counterstain (*gray*) shown in **d**. The *arrows* indicate two Th17 cells double positive for IL-17 and CD3

Irrespective of HPV status, all tumor samples were infiltrated by CD3^+^ T cells, which comprised a substantial population of CD3^+^FoxP3^+^ Tregs (Fig. 1, Supplementary Fig. 1 and Supplementary Table 1). IL-17^+^ cells represented another substantial infiltrating immune cell population, whereas only a minor population of CD3^+^IL-17^+^ Th17 cells was observed. FoxP3^+^ cells were always positive for CD3. FoxP3^+^IL-17^+^ cells were observed very infrequently—at maximum five cells in all samples comprising 0.01 % of FoxP3^+^ cells—and were thus not further analyzed.

**Fig. 2:**
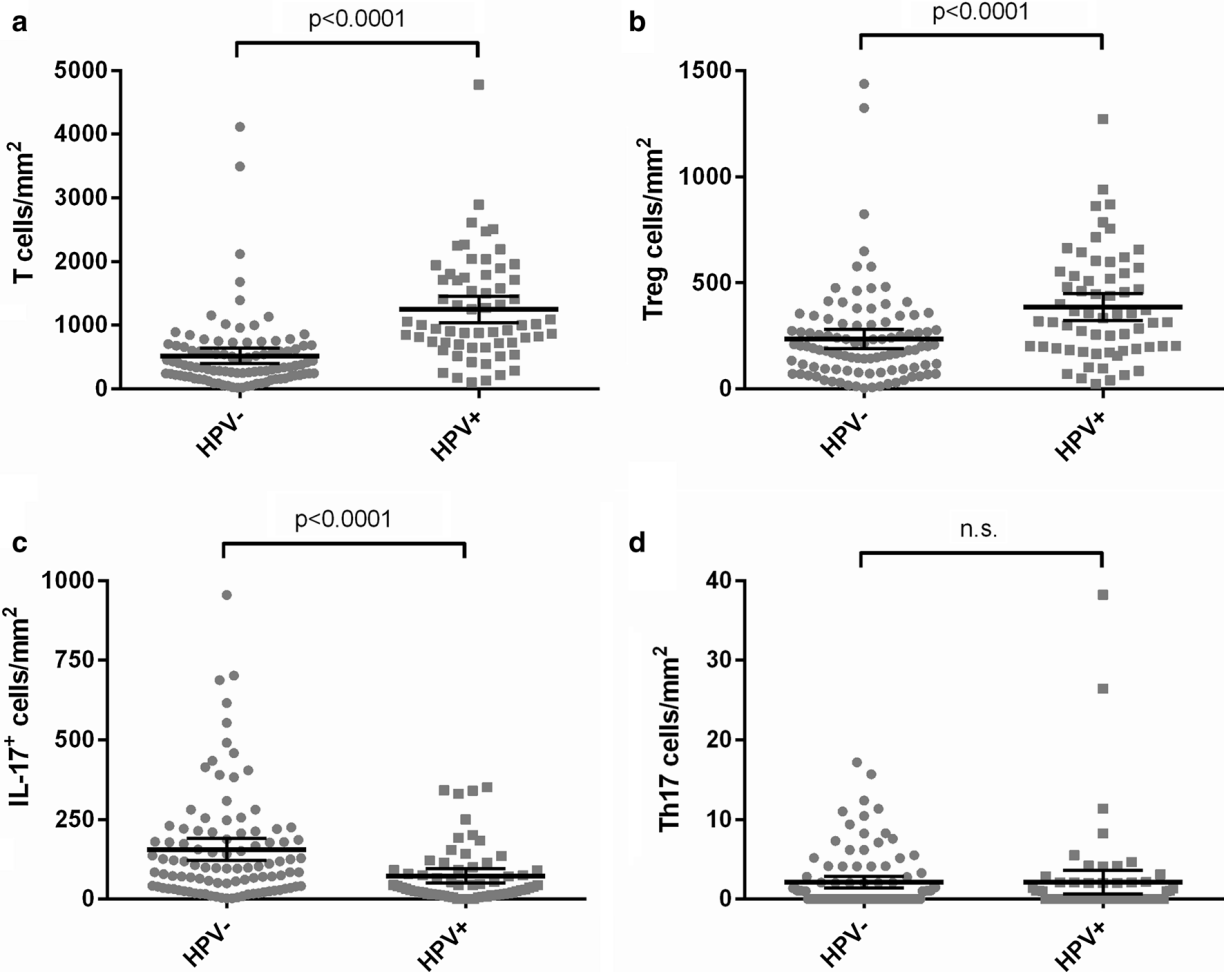
Tumor-infiltrating T cells and IL-17^+^ cells in HPV-positive and HPV-negative tumors. The number of CD3^+^ T cells (**a**), FoxP3^+^CD3^+^ Tregs (**b**), CD3-IL-17^+^ cells (**c**) and CD3^+^IL-17^+^ Th17 cells (**d**) infiltrating in the combined tumor epithelium and stroma per mm^2^ is shown for HPV-negative tumors and HPV-positive tumors. The *bars* indicate the mean and 95 % confidence interval; *n*.*s*. not significant

HPV-positive tumors contained significantly higher numbers of CD3^+^ T cells infiltrating in the tumor epithelium (*p* < 0.0001) and the tumor stroma (*p* < 0.0001). Because the increase in T cells in HPV-positive tumors was similar in the epithelium, stroma and the combined tumor epithelium and stroma field, the significantly increased numbers of CD3^+^ T cells in the HPV-positive compared to the HPV-negative tumors are shown in Fig. 2a for the combined area (*p* < 0.0001). The number of CD3^+^FoxP3^+^ Tregs infiltrating in the tumor epithelium (*p* < 0.0001) and stroma (*p* < 0.0001) was also significantly higher in HPV-positive tumors. The increased number of Tregs in the tumor epithelium and stroma combined in HPV-positive tumors is shown in Fig. 2b (*p* < 0.0001). However, the average ratio of total T cells over Tregs was twice as high in HPV-positive tumors compared to HPV-negative tumors (Supplementary Table 1). Non-Treg T cells were thus particularly increased in HPV-positive tumors. In contrast, the number of IL-17^+^ non-T cells was significantly higher in the tumor epithelium (*p* = 0.003), the stroma (*p* = 0.004) and the tumor epithelium and stroma combined (*p* < 0.0001, Fig. 2c) of HPV-negative compared to HPV-positive tumors. The frequency of Th17 cells was not significantly different between HPV-positive and HPV-negative tumors (Fig. 2d).

The frequency of infiltrating Tregs was significantly correlated with the frequency of total infiltrating T cells in both HPV-positive (*r* = 0.676, *p* < 0.0001) and HPV-negative tumors (*r* = 0.877, *p* < 0.0001). The frequency of infiltrating IL-17^+^ cells was not significantly correlated with the frequency of total infiltrating T cells (data not shown).

### Infiltrating T cells are correlated with improved survival in combination with low IL-17^+^ non-T cell frequencies

**Fig. 3:**
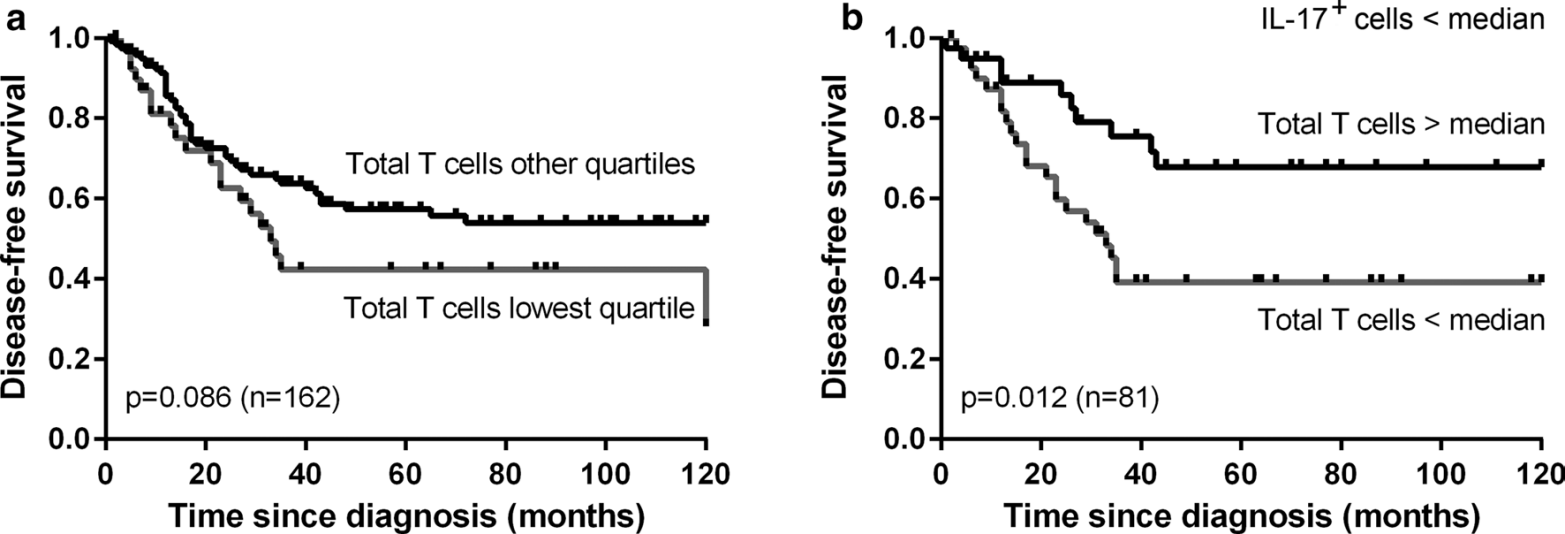
Kaplan–Meier disease-free survival *curves* for a low (i.e., lowest quartile) versus higher number of total T cells among all patients (**a**) and a low (i.e., below median) versus high number of total T cells among the patients with a below median number of IL-17^+^ cells/mm^2^ (**b**) in the tumor epithelium and stroma combined

We subsequently studied the correlations between the infiltrating immune cell frequencies and patient survival. Since the correlations for stromal and total cell numbers were similar, the correlations for intra-epithelial and total cell numbers are discussed. A high number of infiltrating total T cells in all patients combined showed a trend toward correlation with improved disease-specific (*p* = 0.089, data not shown) and disease-free survival (0.086, Fig. 3a) compared to a low number of T cells (i.e., lowest quartile). Previously, we found that cervical cancer-infiltrating IL-17^+^ cells, representing mainly granulocytes, were associated with poor survival [26]. We, therefore, divided the patients based on the median number of IL-17^+^ cells. Among patients with a low number of IL-17^+^ cells, a high number of total infiltrating T cells was correlated with improved disease-specific (*p* = 0.033, data not shown) and disease-free survival (*p* = 0.012, Fig. 3b) when compared to a low T cell frequency. The prognostic effect of tumor-infiltrating T cells was lost in the group of patients with an above number of tumor-infiltrating IL-17^+^ cells (data not shown). Thus, the effect of tumor-infiltrating T cells in OPSCC may be related to the low number of IL-17^+^ cells present.

**Fig. 4:**
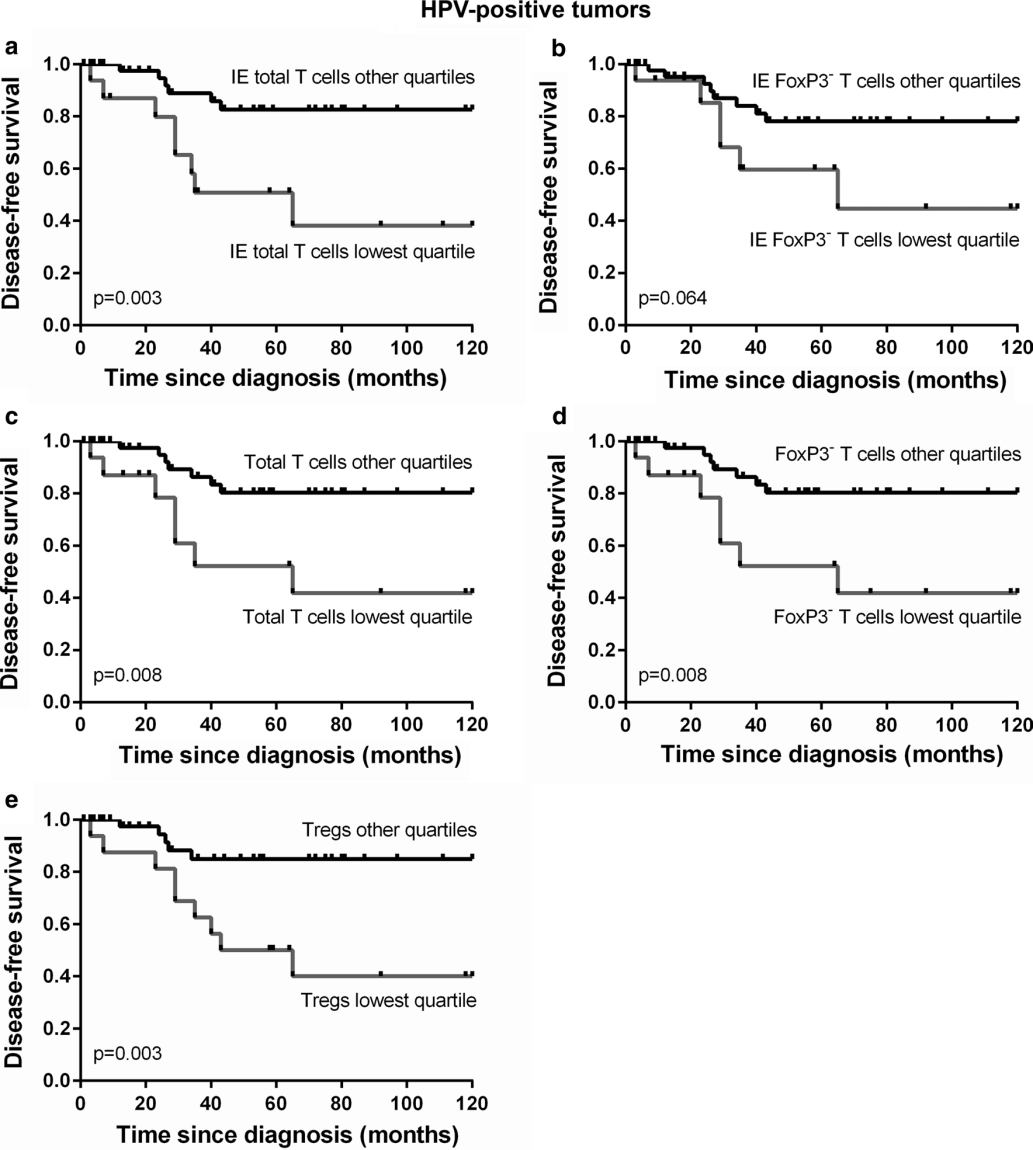
Among patients with HPV-positive tumors (*n* = 63), Kaplan–Meier *curves* are shown for a low versus high number of total T cells (**a**) and non-Treg T cells (**b**) within the tumor epithelium (IE) and a low versus high T cell (**c**), non-Treg T cell (**d**) and Treg (**e**) frequency in the total tumor area (epithelium and stroma combined)

We further studied the survival correlations among patients with HPV-positive tumors. The presence of HPV in OPSCC tumors was significantly correlated with improved disease-specific (*p* = 0.0001) and disease-free survival (*p* < 0.0001, data not shown), corresponding to earlier studies [30, 31]. Since p16-positive tumors were matched to p16-negative tumors for factors that may contribute to prognosis, these factors were equally distributed over the groups of HPV-positive and HPV-negative tumors and similarly correlated with survival. When corrected for comorbidity, prior tumor occurrence and smoking status, the hazard ratio for a recurrence or death to disease with an HPV-positive compared to an HPV-negative tumor was 0.334 (95 % CI: 0.185-0.605, *p* < 0.0001). Analysis of the correlation between tumor-infiltrating immune cells and survival revealed that among patients with HPV-positive tumors, which displayed significantly lower numbers of IL17^+^ cells than the HPV-negative tumors, a high number of intra-epithelial T cells was indeed correlated with improved disease-free survival (*p* = 0.003, Fig. 4a) compared to a low intra-epithelial T cell frequency (i.e., lowest quartile). Similarly, a high non-Treg intra-epithelial T cell frequency showed a trend toward a correlation with improved disease-free survival (*p* = 0.064, Fig. 4b). Furthermore, a high T cell frequency, a high CD3^+^FoxP3^−^ non-Treg T cell frequency and a high Treg frequency infiltrating the total tumor area (epithelium and stroma combined) of HPV-positive tumors were all significantly correlated with improved disease-free survival (*p* = 0.008, *p* = 0.008, *p* = 0.003, respectively; Fig. 4c–e). We also found a trend toward a positive correlation between a high Treg frequency in the total tumor area and disease-specific survival (*p* = 0.055, data not shown). We did not find significant correlations between the IL-17^+^ cell frequencies and disease-free or disease-specific survival among patients with HPV-positive tumors, probably because in most cases the numbers were low when compared to HPV-negative tumors. Only a high intra-epithelial T cell frequency remained significantly correlated with disease-free survival when corrected for comorbidity, prior tumor occurrence and smoking status in a multivariate analysis (Supplementary Table 2).

For patients with HPV-negative tumors, we only found a significant correlation for a high T cell/IL-17^+^ non-T cell ratio and improved disease-specific survival (*p* = 0.043, data not shown). No significant direct correlations between the T cell, Treg or IL-17^+^ cell frequencies and disease-free or disease-specific survival were found (Supplementary Table 2), while the effect of other factors that may contribute to prognosis (comorbidity, prior tumor occurrence and smoking status) remained similar to the effect in patients with HPV-positive tumors (data not shown).

### Epithelium infiltrating T cells in HPV-positive tumors are inversely correlated with smoking status

Because of the correlation described between smoking habits and prognosis in HPV-positive tumors [12], we wondered whether smoking habits may directly influence the tumor infiltration of T cells. Indeed, HPV-positive tumors of heavy smokers (>24 pack-years) were significantly correlated with a lower intra-epithelial T cell frequency compared to tumors of never smokers (*p* = 0.003, Supplementary Fig. 2). The other cell type studies were not significantly correlated with smoking status (data not shown).

### HPV-positive tumor-infiltrating T cells produce IL-17 upon activation

**Fig. 5:**
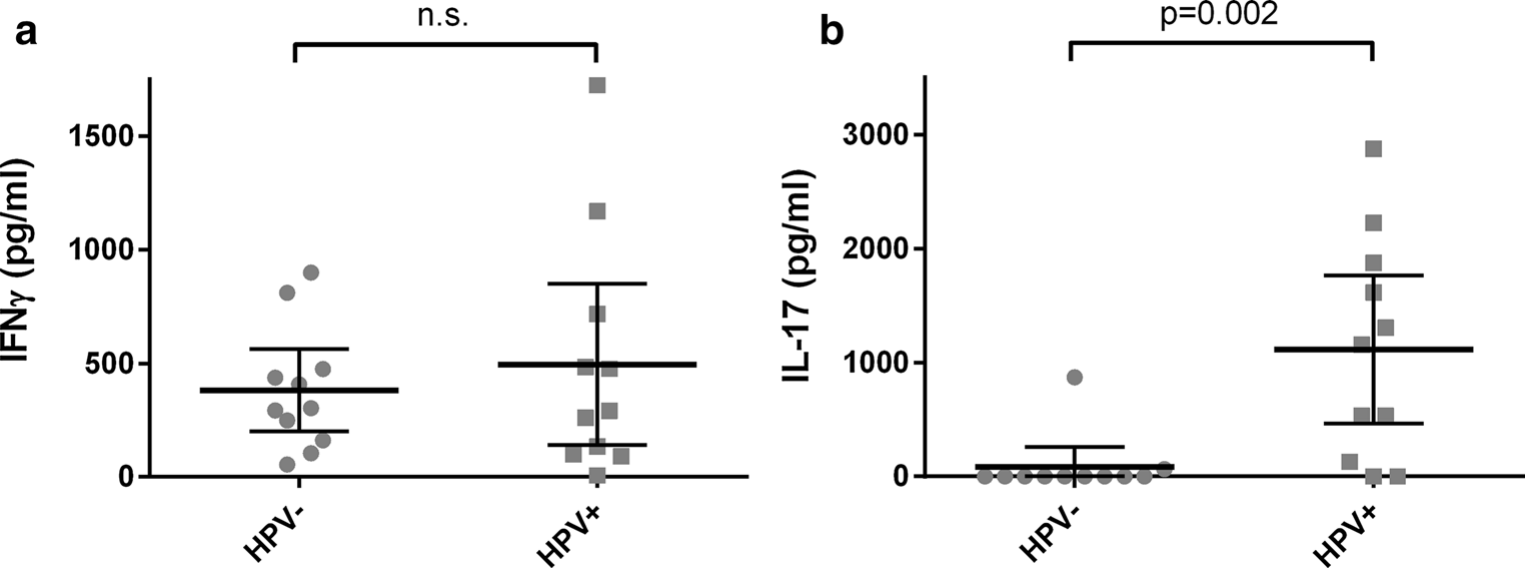
Production of IFN-γ (**a**) and IL-17 (**b**) by tumor-infiltrating lymphocytes stimulated with PHA. The bars indicate the mean and 95 % confidence interval; *n*.*s*. not significant

To study whether the production of effector molecules was influenced by the presence of HPV, we isolated the tumor-infiltrating T cells from 11 HPV-negative OPSCC and 11 HPV-positive OPSCC and assessed cytokine production after 4 days of stimulation with PHA. We studied IFN-γ production as a measure for effector non-Treg T cells, and IL-17 production as a measure for Th17 cells. While IFN-γ was produced in all cases, the TILs isolated from HPV-positive tumors produced IL-17 more frequently (*p* = 0.006) (Fig. 5a, b), suggesting that functional Th17 cells are especially present in HPV-positive tumors.

## Discussion

HPV-positive OPSCC contained more tumor-infiltrating T cells and less IL-17^+^ non-T cells compared to HPV-negative tumors in both the epithelial and stromal part of the tumor. An increased number of CD3^+^, CD8^+^ and Treg cells [32–34] and a trend toward a decreased number of IL-17^+^ cells [35] infiltrating HPV-positive compared to HPV-negative OPSCC have been shown previously [36]. Although correlations between a high tumor-infiltrating lymphocyte frequency and improved survival in both patients with HPV-positive [37] and HPV-negative tumors [16, 33, 38] have been described before, data regarding the T cell subtypes involved have been limited and inconclusive. The current study revealed that a high number of intra-tumoral T cells showed a trend toward better survival of all (HPV-positive and HPV-negative) OPSCC patients. Since we have shown before that a high frequency of IL-17^+^ non-T cells, representing mainly granulocytes is correlated with poor survival in early-stage squamous cervical cancer [26], here we studied the effect of tumor-infiltrating T cells stratified for a high or low number of infiltrating IL-17^+^ cells. In patients with a below median number of intra-tumoral IL-17^+^ non-T cells, a high tumor-infiltrating T cell frequency was correlated with improved disease-free and disease-specific survival, suggesting that a high frequency of IL-17^+^ cells is related to a poor immune response. No significant correlation was observed in tumors with a high number of IL-17^+^ non-T cells. The hypothesis was further substantiated by the observation that in the HPV-positive OPSCC, which contained less IL-17^+^ cells than HPV-negative OPSCC, a high number of T cells was correlated with improved disease-free survival. This suggests that IL-17^+^ non-T cells may be correlated with an unfavorable immune response. Such a tumor-promoting role can be explained by the role of IL-17 in driving inflammation, angiogenesis and tumor growth, and studies so far have indeed described correlations between IL-17 and poor survival in cancer patients [27]. Thus, the beneficial effect of infiltrating T cells might be overruled if a high number of IL-17^+^ cells are present.

Among patients with HPV-positive tumors, we specifically found correlations with improved disease-free survival for high frequencies of both non-Treg T cells and Tregs. A high number of Tregs also showed a trend toward a correlation with improved disease-specific survival in HPV-positive OPSCC. The role of Tregs is controversial in OPSCC [16]. We have shown before that a high T cell infiltration in cervical cancer is correlated with improved prognosis [39], with specifically a low T cell/Tregs ratio within the tumor epithelium being correlated with poor survival [40, 41]. Indeed, only a high intra-epithelial total T cell frequency remained significantly correlated with disease-free survival in the multivariate analyses performed here. Because we now show that the intra-tumoral Treg frequency was increased and strongly correlated with the total T cell frequency in a ratio that favors the infiltration of non-Treg T cells in HPV-positive OPSCC, the positive role of Tregs in oropharyngeal cancer may also rely on their co-infiltration with effector T cells. The current data suggest that a high T cell infiltrate, including Tregs, is correlated with improved prognosis in HPV-positive OPSCC.

A minor Th17 cell population was observed, which was not significantly different between HPV-positive and HPV-negative tumors. However, we showed that T cells infiltrating HPV-positive tumors produced significantly higher amounts of IL-17 compared to T cells infiltrating HPV-negative tumors. This activated state may be an indication that Th17 cells are associated with a tumor-targeting immune response. In agreement, Partlová et al. [34] also showed that cell suspensions prepared from HPV-positive head and neck squamous cell carcinoma produced more IL-17 than cell suspensions from HPV-negative tumors. These data together strongly suggest that Th17 cells are more active in HPV-positive tumors. The seemingly opposing small population size and large potential of Th17 cells might be explained by their stem cell-like phenotype [42] and potential for plasticity [43]. This corresponds with the correlations described between Th17 cells and improved cancer patient survival [27], including our study in squamous cervical cancer [26].

We did not find any direct correlations between the infiltrating immune cell frequencies investigated and disease-free or disease-specific survival in HPV-negative tumors. Only the T cell/IL-17^+^ non-T cell ratio was significantly correlated with disease-specific survival, again suggesting that the beneficial effect of T cells may be lost because of the higher numbers of IL-17^+^ non-T cells present in HPV-negative OPSCC.

To conclude, HPV-positive OPSCC contain higher numbers of tumor-infiltrating T cells, more active Th17 cells and lower numbers of IL-17^+^ non-T cells. Future studies should evaluate whether this is a general signature of a beneficial tumor-targeting immune response. This would provide a rationale to study the role and potential of T cell administration or IL-17 blockade.

### Electronic supplementary material

Below is the link to the electronic supplementary material.

Supplementary material 1 (PDF 347 kb)

## Abbreviations

DAB: 3,3′-Diamino-benzidine-tetrahydrochloride
FFPE: Formaldehyde fixed, paraffin embedded
IE: Intra-epithelial
LUMC: Leiden University Medical center
ONCDOC: Oncology Documentation
OPSCC: Oropharyngeal squamous cell carcinoma
Th17 cell: T helper 17 cell
Tregs: Regulatory T cells
VUMC: VU Medical Center
