# Supplementary material for: A beneficial tumor microenvironment in oropharyngeal squamous cell carcinoma is characterized by a high T cell and low IL-17+ cell frequency
Source: Cancer Immunol Immunother. 2016 Feb 22;65(4):393–403. doi: 10.1007/s00262-016-1805-x (PMC4826411; doi:10.1007/s00262-016-1805-x)
Supplement: Supplementary file 1 — Supplementary material 1 (PDF 347 kb) [file 262_2016_1805_MOESM1_ESM.pdf]

Supplementary Figure 1. Quantification of tumor infiltrating cells

a

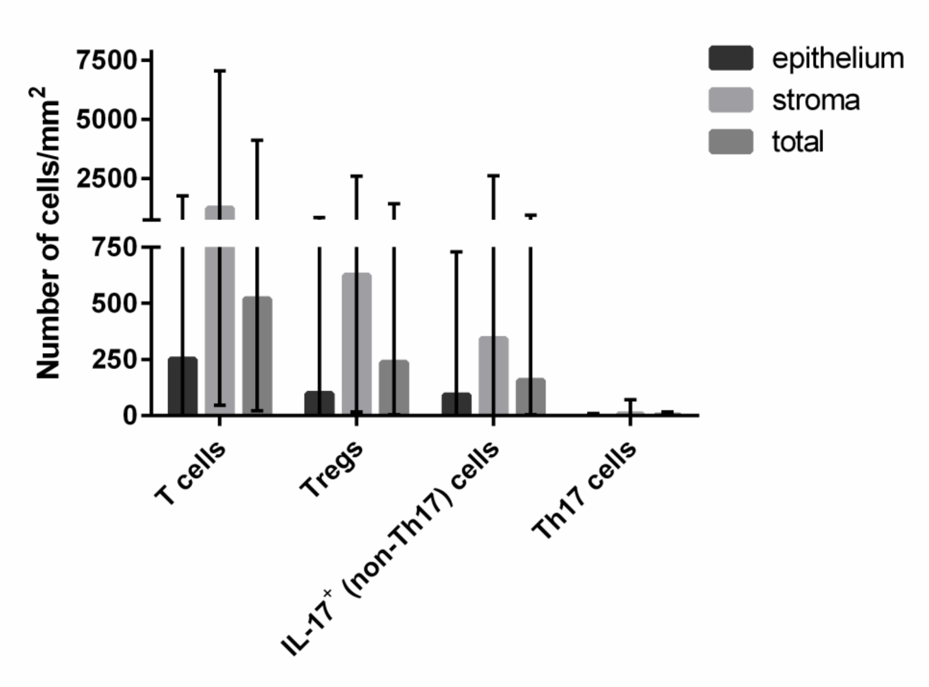

b

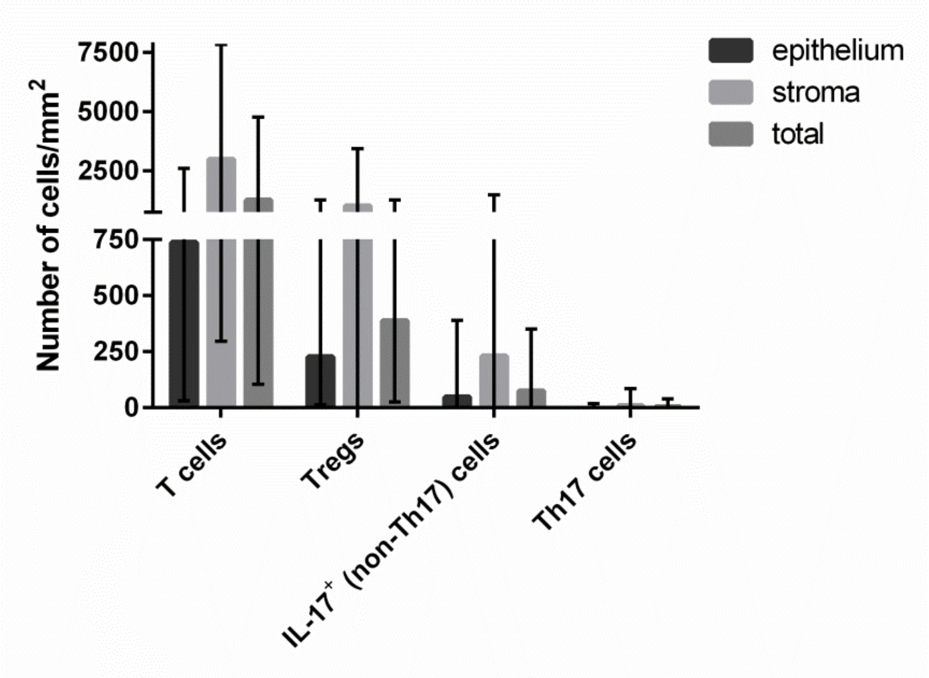

The mean and range of the number of $CD3^+$ T cells, $FoxP3^+CD3^+$ Tregs, $CD3^-IL\text{-}17^+$ cells and $CD3^+IL\text{-}17^+$ Th17 cells infiltrating in the tumor epithelium, tumor stroma and combined total area per mm^2 is shown in HPV-negative tumors (**a**, n=99) and HPV-positive tumors (**b**, n=63).

Supplementary Figure 2. Decreased intraepithelial T-cell frequency in HPV-positive OPSCC of heavy compared to never smokers

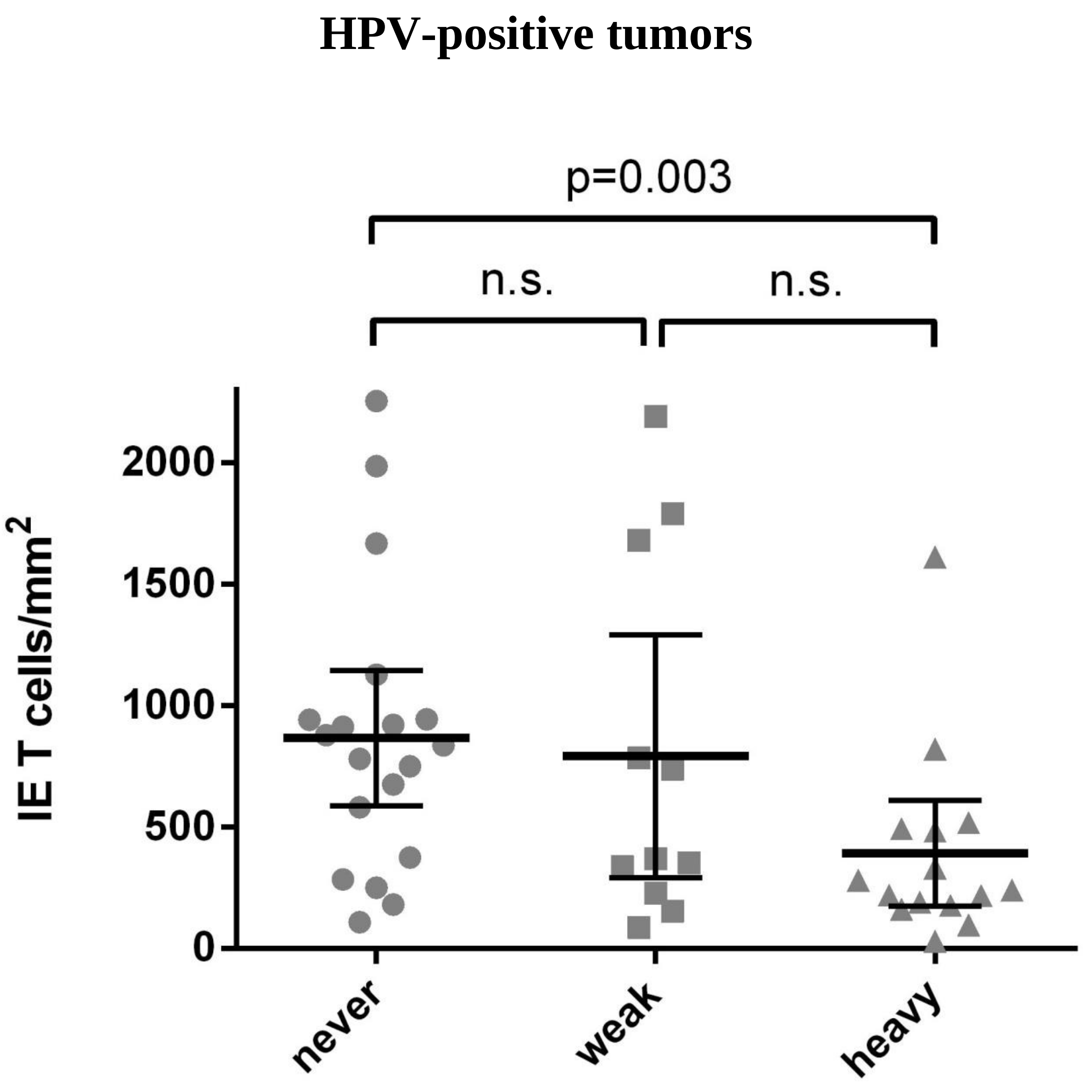

The number of total $CD3^{+}$ T cells/mm^2 tumor epithelium in HPV-positive tumors is shown for never, weak (1-24 packyears) and heavy smokers (>24 packyears). The bars indicate the mean and 95% confidence interval; n.s. = not significant.

Supplementary Table 1. Number of T cells, Tregs and IL-17$^+$ cells present in squamous oropharyngeal carcinoma

	Location	Total T cells		Tregs		IL-17$^+$ (non-Th17) cells		Th17 cells		Total T cells/Tregs	
		Mean (range)	Median	Mean (range)	Median	Mean (range)	Median	Mean (range)	Median	Mean (range)	Median
HPV-negative tumors	Epithelium	251 (0-1767)	151	98 (0-863)	49	92 (0-730)	41	1 (0-9)	0	4 (1-37)	2
	Stroma	1237 (47-7042)	971	624 (16-2596)	520	343 (0-2613)	237	6 (0-70)	0	2 (1-8)	2
	Total	519 (21-4113)	362	237 (4-1438)	193	156 (3-956)	99	2 (0-17)	0	2 (1-8)	2
HPV-positive tumors	Epithelium	738 (30-2596)	545	226 (12-1272)	150	47 (0-389)	14	1 (0-17)	0	6 (1-84)	3
	Stroma	2985 (295-7831)	2633	1017 (0-3437)	972	229 (0-1479)	121	7 (0-85)	0	4 (1-35)	3
	Total	1251 (104-4775)	1005	387 (24-1272)	335	73 (0-351)	39	2 (0-38)	0	5 (1-63)	3

The mean, minimum, maximum and median number of total CD3$^+$ cells, FoxP3$^+$CD3$^+$ Tregs, CD3$^-$IL-17$^+$ cells and CD3$^+$IL-17$^+$ Th17 cells observed infiltrating in the tumor epithelium, tumor stroma and combined total area per mm^2 is indicated for HPV-negative and HPV-positive tumors.

Supplementary Table 2. Effect of 6 different types of T cell infiltrations on disease-free survival regarding HPV status

	High intraepithelial T cell frequency		High total T cell frequency		High total Treg frequency		High total nonTreg T cell frequency		High intraepithelial nonTreg T cell frequency		High total IL-17^{+} cell frequency	
**Univariate Hazard Ratio**	*HR (95%CI)*	*p-value*	*HR (95%CI)*	*p-value*	*HR (95%CI)*	*p-value*	*HR (95%CI)*	*p-value*	*HR (95%CI)*	*p-value*	*HR (95%CI)*	*p-value*
HPV negative (p16-)	1.46 (0.73-2.93)	0.282	1.11 (0.58-2.12)	0.751	1.65 (0.81-0.36)	0.165	1.62 (0.81-3.26)	0.174	1.15 (0.89-1.50)	0.292	0.96 (0.51-1.82)	0.912
HPV positive (p16+/PCR+)	0.22 (0.08-0.64)	**0.006**	0.28 (0.10-0.82)	**0.021**	0.21 (0.07-0.64)	**0.006**	0.27 (0.09-0.78)	**0.015**	0.35 (0.12-1.03)	0.056	0.50 (0.17-1.51)	0.220
**Multivariate Hazard Ratio***	*HR (95%CI)*	*p-value*	*HR (95%CI)*	*p-value*	*HR (95%CI)*	*p-value*	*HR (95%CI)*	*p-value*	*HR (95%CI)*	*p-value*	*HR (95%CI)*	*p-value*
HPV negative (p16-)	1.52 (0.74-3.18)	0.256	1.13 (0.57-2.21)	0.721	1.77 (0.83-3.74)	0.134	1.66 (0.81-3.41)	0.168	1.16 (0.84-1.88)	0.258	1.09 (0.54-2.18)	0.806
HPV positive (p16+/PCR+)	0.25 (0.07-0.95)	**0.043**	0.34 (0.10-1.13)	0.080	0.28 (0.08-1.02)	0.054	0.40 (0.12-1.32)	0.135	1.18 (0.24-5.97)	0.835	0.53 (0.17-1.69)	0.284

*Adjusted for comorbidity, prior tumor and smoking status using Cox proportional hazards regression analysis. This analysis was performed 6 times, entering a different type of T cell infiltration each time, but adjusting for the same variables (comorbidity, prior tumor and smoking status).

HR= hazard ratio, 95%CI = 95% confidence interval. 'High'= upper 3 qrt vs lowest qrt.
